# Simulation‐Based Bayesian Predictive Probability of Success for Interim Monitoring of Clinical Trials With Competing Event Data: Two Case Studies

**DOI:** 10.1002/pst.70050

**Published:** 2025-11-25

**Authors:** Chiara Micoli, Alessio Crippa, Jason T. Connor, I‐SPY COVID Consortium, Martin Eklund, Andrea Discacciati

**Affiliations:** ^1^ Department of Medical Epidemiology and Biostatistics Karolinska Institutet Stockholm Sweden; ^2^ ConfluenceStat LLC Cooper City Florida USA; ^3^ University of Central Florida College of Medicine Orlando Florida USA

**Keywords:** Bayesian predictive probability of success, clinical trials, competing events, interim analysis

## Abstract

Bayesian predictive probabilities of success (PPoS) use interim trial data to calculate the probability of trial success. These quantities can be used to optimise trial size or to stop for futility. In this paper, we describe a simulation‐based approach to compute the PPoS for clinical trials with competing event data, for which no specific methodology is currently available. The proposed procedure hinges on modelling the joint distribution of time to event and event type by specifying Bayesian models for the cause‐specific hazards of all event types. This allows the prediction of outcome data at the conclusion of the trial. The PPoS is obtained by numerically averaging the probability of success evaluated at fixed parameter values over the posterior distribution of the parameters. Our work is motivated by two randomised clinical trials: the I‐SPY COVID phase II trial for the treatment of severe COVID‐19 (NCT04488081) and the STHLM3 prostate cancer diagnostic trial (ISRCTN84445406), both of which are characterised by competing event data. We present different modelling alternatives for the joint distribution of time to event and event type and show how the choice of the prior distributions can be used to assess the PPoS under different scenarios. The role of the PPoS analyses in the decision‐making process for these two trials is also discussed.

**Abbreviations**
DMC: data monitoring committee
HR: hazard ratio
PCa: prostate cancer
PCH: piecewise constant cause‐specific hazard
PCM: prostate cancer mortality
PPoS: predictive probability of success
PSA: prostate‐specific antigen
RR: risk ratio

## 1 Introduction

The interim monitoring of clinical trials has become an essential component of trial design [1]. Bayesian methods are particularly well suited for this purpose, as they enable the prediction of the probability of trial success at its conclusion based on interim data, averaged over the posterior distribution of the treatment effect and other nuisance parameters [2]. By predicting the probability that the trial will provide sufficient evidence to support the study's primary hypothesis if the trial continues to its end, the Bayesian predictive probability of success (PPoS) can inform decisions regarding the continuation, modification, or early termination of the trial. If the PPoS is too low, and the trial is therefore predicted to be unlikely to achieve its objective, the trial may be stopped early for futility [3, 4, 5], reducing the risk of exposing participants to ineffective or harmful therapies and conserving resources that can be reallocated to more promising trials [1]. Conversely, if completing the prespecified follow‐up period for participants who have already been enrolled is predicted to lead to success with a sufficiently high probability, enrolment of new participants can be stopped, and the primary outcome can be analysed at the conclusion of the follow‐up period [3, 6]. Furthermore, the PPoS can be employed to adjust the sample size at interim analyses [7] or to inform the timing of the primary analysis, by determining the minimum duration of the follow‐up period required to achieve a certain PPoS [8].

Predictive probabilities of success have been primarily employed to monitor clinical trials with continuous or binary endpoints, e.g., in the I‐SPY 2 trial [9] and the REST trial [10]. In contrast, the use of PPoS in clinical trials with survival data is considerably less common [6, 11, 12]. One possible approach to deriving PPoS relies on asymptotic approximations of the posterior distribution or the test statistic for the parameter of interest, as well as the approximation of the information fraction at interim analysis [13, 14, 15]. PPoS based on these asymptotic arguments has been compared with those based on more flexible and general simulation‐based approaches [13, 14, 16], also referred to as imputed predictive probabilities [15]. Results showed that the PPoS computed using these approximations do not always align with the corresponding simulation‐based PPoS. In fact, simulation may still be necessary to assess the performance of approximation‐based PPoS [15]. Existing simulation‐based methods for survival data are limited to those settings where the endpoint is defined as the time span from randomisation until the occurrence of a single event type [7, 13, 14, 17, 18]. Furthermore, these approaches adopt simple Bayesian survival models for the distribution of the time‐to‐event outcome, such as exponential [7, 13, 17] or piecewise exponential models [14], and leverage conjugate priors to derive closed‐form posterior distributions. While these choices allow for tractable analytical solutions, they limit the flexibility and applicability of these methods. In these simulation‐based approaches, the follow‐up times for the patients still at risk at interim monitoring are censored and these data contribute to the fit of the Bayesian survival model. The predicted survival times for these patients are then sampled from the posterior predictive distribution. To the best of our knowledge, no specific methodology has been proposed to account for competing event data when computing the PPoS.

To fill this gap, in this paper we take the approach of computing PPoS using statistical simulation [19] and extend it to the setting of survival data with competing events. Competing events occur frequently in survival data from clinical trials, where participants may experience different types of events and the occurrence of one event precludes the occurrence of all other event types [20]. For example, in randomised clinical trials evaluating potential treatments for hospitalised patients diagnosed with COVID‐19, hospital discharge due to recovery and death without prior recovery are mutually exclusive (i.e., competing) events [21]. We propose to model the joint distribution of time to event and event type by specifying parametric Bayesian models for the cause‐specific hazard functions, allowing the prediction of the outcome data at the conclusion of the trial and thus the computation of the PPoS. Our suggested procedure provides a general approach that can be used for interim monitoring of newly designed trials or to extend existing designs, for example for phase II [22] or phase III clinical trials [7], to competing event data.

Our work is motivated by two randomised clinical trials: the I‐SPY COVID phase II trial for the treatment of severe COVID‐19 [23] (NCT04488081) and the STHLM3 prostate cancer diagnostic trial [24] (ISRCTN84445406). Both trials are characterised by a time‐to‐event outcome, namely time to recovery from COVID and time to prostate cancer mortality. In the I‐SPY COVID trial, death from any cause is a competing event and the PPoS was used at interim monitoring to inform the decision to continue randomisation of patients to a specific investigational agent. In the STHLM3 trial, death from causes other than prostate cancer is a competing event and the PPoS was employed to predict the shortest follow‐up period necessary to accumulate enough prostate cancer deaths in order to reach sufficient power for the final analysis.

## 2 Methods

### 2.1 Notation and Preliminaries

For each trial participant s, where s=1,…,N, let $T_{s}$ be the absolutely continuous variable representing the true event time with support on the positive real half‐line, and let $X_{s}\in \left\{1,\ldots ,I\right\}$ be the competing event indicator. The right‐censored event time is denoted by $Y_{s}=\min\left(T_{s},C_{s}\right)$, where $C_{s}$ is the censoring time, and $V_{s}=1\left(T_{s}\leq C_{s}\right)\cdot X_{s}\in \left\{0,1,\ldots ,I\right\}$ is the observed event indicator, where 0 indicates right censoring, and 1(·) is the indicator function. Lastly, let $Z_{s}$ be a vector of baseline covariates, including at least the randomisation arm $A_{s}$. In total, we define the random vector $D_{s}=\left(Y_{s},V_{s},Z_{s}\right)$. Hereafter, we suppress the subscript s to simplify notation.

The cause‐specific hazard for the i‐th competing event is defined as

$$\lambda_{i}\left(t\right)=\lim_{\mathrm{dt}↓0}\frac{\mathrm{Pr}\left(T\leq t+\mathrm{dt},X=i\;|\;T>t\right)}{\mathrm{dt}}.$$

The cause‐specific hazards for all events collectively define the joint distribution of $\left(T,X\right)$ [25]. The crude cumulative probabilities (risks) of experiencing the competing event i by the time t are defined as $F_{i}\left(t\right)=\mathrm{Pr}\left(T\leq t,X=i\right)=\int_{0}^{t}\lambda_{i}\left(u\right)\;\exp\left(-\sum_{i=1}^{I}\int_{0}^{u}\lambda_{i}\left(s\right)\mathrm{ds}\right)\mathrm{du}$.

At interim monitoring, the goal is to predict the probability of trial success at the final analysis given the interim data and the assumed prior distributions on the parameter vector θ for the data‐generating process used to predict future data. Let $d_{0}$ be the observed data at the time of interim analysis, $D_{f}$ be the future data available only at the time of final analysis, which encompasses both observed and yet‐to‐be‐observed data, and let R be the critical region such that if $D_{f}\in R$ the trial will be declared a success. The PPoS, introduced by Spiegelhalter et al. [2], is defined as:

$$\begin{array}{c} \text{PPoS}=\mathrm{Pr}\left(D_{f}\in R\;|\;d_{0}\right)=\int \mathrm{Pr}\left(D_{f}\in R\;|\;\theta ,d_{0}\right)p\left(\theta \;|\;d_{0}\right)\mathrm{d\theta}\\ \;=E_{\theta |d_{0}}\left[E\left[1\left(D_{f}\in R\right)\;|\;\theta ,d_{0}\right]\right]. \end{array}$$

In the PPoS, the conditional power $\mathrm{Pr}\left(D_{f}\in R\;|\;\theta ,d_{0}\right)$, defined as the probability of trial success given the interim data and a fixed θ, is averaged over the posterior distribution of θ, denoted $p\left(\theta \;|\;d_{0}\right)$. The definition of trial success can be based on Bayesian posterior probabilities, as in the I‐SPY COVID trial, or on frequentist *p*‐values, as in the STHLM3 trial [3].

Three possible types of data must be considered when computing the PPoS at interim monitoring with time‐to‐event data. Firstly, there are completely observed data, where the event type, the time to the event, and the baseline covariates are known ($d_{\mathrm{obs}}=\left(t,x,z\right)$). Secondly, there are partially observed data, where the baseline covariates are known, but the follow‐up time is right‐censored at the time of interim monitoring ($d_{\text{cens}}=\left(c,0,z\right)$). Thirdly, there are completely unobserved data, which will be observed if new participants are enrolled in the future ($D_{\mathrm{new}}=\left(Y,V,Z\right)$). In order to relate these types of data to the PPoS formula, note that $d_{0}$ is given by stacking $d_{\mathrm{obs}}$ and $d_{\text{cens}}$ ($d_{0}=\left(d_{\mathrm{obs}},d_{\text{cens}}\right)$). The data $D_{f}$ include $d_{\mathrm{obs}}$, $D_{\mathrm{new}}$, but also $D_{\text{cens}}=\left(Y,V,z\right)$, representing the updated data at the conclusion of the trial for the participants in $d_{\text{cens}}$ ($D_{f}=\left(d_{\mathrm{obs}},D_{\text{cens}},D_{\mathrm{new}}\right)$). At interim monitoring, $D_{\mathrm{new}}$ and $D_{\text{cens}}$ are not fully observed and to calculate the simulation‐based PPoS they need to be predicted from statistical models.

### 2.2 Predictive Probability of Success With Competing Event Data

The framework to predict the probability of trial success via statistical simulation can be divided into three phases: modelling, prediction, and analysis [19]. The proposed approach specialises the first and second phases to allow the modelling and prediction of competing event data, which are then analysed in the third phase. In this section, we describe each step of the simulation process and summarise it in a compact form in Algorithm 1.

#### 2.2.1 Modelling Phase

The goal of the modelling phase is to specify a Bayesian model for the joint distribution of the competing event data, the baseline data, and the parameters [26]:

(1) $$p\left(t,x,z,\theta \right)=p\left(t,x,z\;|\;\theta \right)p\left(\theta \right).$$

To facilitate modelling, we decompose $p\left(t,x,z\;|\;\theta \right)$ into a product of two distributions: one for the competing event data given the baseline data and one for the baseline data. We assume that these two distributions have no shared parameters and write $\theta =\left(\xi ,\eta \right)$. We also assume that the priors on the parameters of the two distributions are independent, i.e., that $p\left(\theta \right)=p\left(\xi \right)p\left(\eta \right)$. As a result, the joint distribution in Equation (1) can be rewritten as follows:
(2)

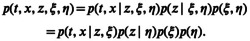




We propose to specify the distribution for the competing event data in terms of cause‐specific hazards for all I event types [27]:

$$p(t,x|z,\xi )=\left(S(t|z,\xi )\sum_{i=1}^{I}\lambda_{i}(t|z,\xi_{i})\right)\prod_{i=1}^{I}{\left(\frac{\lambda_{i}(t|z,\xi_{i})}{\sum_{i}\lambda_{i}(t|z,\xi_{i})}\right)}^{1(x=i)}$$

where $S\left(t\;|\;z,\xi \right)=\exp\left(-\sum_{i=1}^{I}\int_{0}^{t}\lambda_{i}\left(u\;|\;z,\xi_{i}\right)\mathrm{du}\right)$ is the event‐free survival function. Assuming an independent right‐censoring mechanism, the contribution to likelihood for the s‐th observation is proportional to [25]:

$$S\left(y_{s}\;|\;z_{s},\xi \right)\prod_{i=1}^{I}\lambda_{i}{\left(y_{s}\;|\;z_{s},\xi_{i}\right)}^{1\left(v_{s}=i\right)}.$$

Patients censored at the interim analysis ($d_{\text{cens}}$) are still at risk of experiencing any of the competing events. They contribute to the likelihood with the event‐free survival function evaluated at their censoring time.

The model for the distribution of the baseline data depends on the specific type of data to be modelled. The contribution to likelihood for the s‐th observation is written generically as:

$$p\left(z_{s}\;|\;\eta \right).$$

Lastly, the prior distributions on the model parameters, $p\left(\xi \right)$ and $p\left(\eta \right)$, complete the specification of the joint distribution in Equation (2).

The model is then fitted to the interim data $d_{0}$ and samples are drawn from the posterior distribution:

$$p\left(\xi ,\eta \;|\;d_{0}\right)\propto p\left(\xi \right)p\left(\eta \right)\prod_{s=1}^{N}p\left(z_{s}\;|\;\eta \right)S\left(y_{s}\;|\;z_{s},\xi \right)\prod_{i=1}^{I}\lambda_{i}{\left(y_{s}\;|\;z_{s},\xi_{i}\right)}^{1\left(v_{s}=i\right)},$$

for example, using Markov Chain Monte Carlo (MCMC) machinery.

#### 2.2.2 Prediction Phase

In this phase, predictions for future observations are made by sampling from the posterior predictive distribution [26]:

$$\begin{array}{l} p\left(\tilde{t},\tilde{x},\tilde{z}\;|\;d_{0}\right)=\int \int p\left(\tilde{t},\tilde{x},\tilde{z}\;|\;\xi ,\eta \right)p\left(\xi ,\eta \;|\;d_{0}\right)\text{dξdη}\\ =\int \int p\left(\tilde{t},\tilde{x}\;|\;\tilde{z},\xi \right)p\left(\tilde{z}\;|\;\eta \right)p\left(\xi ,\eta \;|\;d_{0}\right)\text{dξdη}. \end{array}$$

As the posterior predictive distribution is not available in closed form, posterior predictive samples can be generated by first decomposing $p\left(t,x\;|\;z,\xi \right)$ into the product $p\left(t\;|\;z,\xi \right)p\left(x\;|\;t,z,\xi \right)$ and then employing the following forward sampling algorithm:

1. Make a draw from the posterior distribution:

$$\left(\tilde{\xi },\tilde{\eta }\right)∼p\left(\xi ,\eta \;|\;d_{0}\right).$$

2 Simulate the baseline covariate data for the future trial participants, if any:

$$\tilde{z}∼p\left(z\;|\;\tilde{\eta }\right).$$

3 Simulate the event times:

- For future trial participants:

$$\tilde{t}∼p\left(t\;|\;\tilde{z},\tilde{\xi }\right)=S\left(t\;|\;\tilde{z},\tilde{\xi }\right)\sum_{i=1}^{I}\lambda_{i}\left(t\;|\;\tilde{z},{\tilde{\xi }}_{i}\right).$$

Event times can be simulated by applying the probability integral transformation [28]. First, draw a random value $\tilde{u}∼\text{Uniform}\left(0,1\right)$ and then generate an event time as $\tilde{t}=S^{-1}\left(\tilde{u}\;|\;\tilde{z},\tilde{\xi }\right)$. If the event‐free survival function cannot be inverted analytically, root finding is used to numerically solve $S\left(\tilde{t}\;|\;\tilde{z},\tilde{\xi }\right)=\tilde{u}$ for $\tilde{t}$.

b For participants censored at the time of interim analysis:

$$\tilde{t}∼p\left(t\;|\;t>c,z,\tilde{\xi }\right)=\frac{p\left(t\;|\;z,\tilde{\xi }\right)1\left(t>c\right)}{1-\int_{0}^{c}p\left(u\;|\;z,\tilde{\xi }\right)\mathrm{du}}.$$

In this case, the event times are simulated conditionally on being still at risk at the observed censoring time c and given the observed baseline data z. This is done by treating c as a left‐truncation time and simulating the event times from the corresponding truncated distribution, where $p\left(t\;|\;z,\tilde{\xi }\right)$ is defined as $S\left(t\;|\;z,\tilde{\xi }\right)\sum_{i=1}^{I}\lambda_{i}\left(t\;|\;z,{\tilde{\xi }}_{i}\right)$.

4 Simulate the event types:

- For future trial participants:

$$\tilde{x}∼p\left(x\;|\;\tilde{t},\tilde{z},\tilde{\xi }\right),$$

where $p\left(x\;|\;t,z,\xi \right)$ is a $\text{Multinomial}\left(1,\left(\pi_{1},\ldots ,\pi_{I}\right)\right)$ distribution with $\pi_{i}=\mathrm{Pr}\left(X=i\;|\;\tilde{t},\tilde{z},\tilde{\xi }\right)=\frac{\lambda_{i}\left(\tilde{t}\;|\;\tilde{z},{\tilde{\xi }}_{i}\right)}{\sum_{i}\lambda_{i}\left(\tilde{t}\;|\;\tilde{z},{\tilde{\xi }}_{i}\right)}$ [29].

b For participants censored at the time of interim analysis:

$$\tilde{x}∼p\left(x\;|\;\tilde{t},z,\tilde{\xi }\right),$$

where the conditioning is on the observed baseline data z, instead of the predicted data $\tilde{z}$.

Lastly, generate censoring times (e.g., at the maximum allowed follow‐up time) and compute $\tilde{y}$ and $\tilde{v}$.

Note that if no further participants are to be recruited after interim monitoring, the Prediction phase is simplified because sampling from $p\left(z\;|\;\tilde{\eta }\right)$ is unnecessary. The Modelling phase is also simplified, as modelling the distribution $p\left(t,x,\xi \;|\;z\right)$ is sufficient.

#### 2.2.3 Analysis Phase

The data completely observed at the time of interim monitoring and the data generated in the Prediction phase are stacked into $D_{f}$ and analysed using the prespecified frequentist or Bayesian analytical method, $g\left(\cdot \right)$, which maps $D_{f}$ to the space where the critical region R is defined. The binary variable G indicating whether the simulated trial reaches success at final analysis is defined as:

$$G=1\left(g\left(D_{f}\right)\in R\;|\;\tilde{\xi },\tilde{\eta }\right).$$

To predict the probability of trial success, the Prediction and Analysis phases are repeated a sufficiently large number of times, and the value $G^{\left(k\right)}$ is recorded for each iteration k=1,…,K. The proportion of simulated trials that achieve success approximates the PPoS [19]:

$$\text{PPoS}\approx \frac{1}{K}\sum_{k=1}^{K}G^{\left(k\right)}.$$

### 2.3 Software

All the analyses were performed in R version 4.3.2 [30]. Bayesian models were fitted to the data in Stan version 2.32.6 using full Bayesian statistical inference with MCMC sampling [31]. The brms package version 2.21.0 was used as the interface to Stan [32]. For Bayesian models with Weibull hazard, we used a custom response distribution available at https://github.com/anddis/brms‐weibullPH. The non‐parametric Aalen–Johansen estimates of the cumulative incidence functions were obtained using the survival package version 3.7–0 [33]. We provide the R code for reproducing the analyses on a synthetic dataset at https://github.com/cmicoli/PPoS‐CompetingRisks.

ALGORITHM 1 **Simulation‐Based Algorithm to Approximate the Bayesian Predictive Probability of Success (PPoS) in Trials With Competing Event Data.**

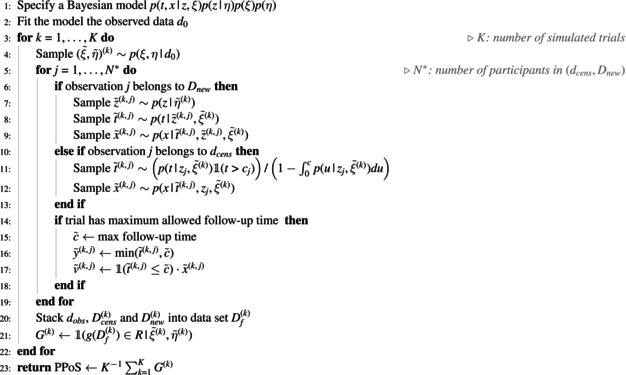

*Note:* T denotes the true event time; X is the competing event indicator; Z is a vector of baseline covariates; ξ and η are the model parameters; k=1,…,K indicates the simulated trials; $j=1,\ldots ,N^{*}$ indexes the observations to be predicted at final analysis; $d_{0}$ is the data observed at interim analysis; $d_{\text{cens}}$ denotes the set of participants censored at interim analysis; and $D_{\mathrm{new}}$ represents the set of future participants enrolled in the trial.

## 3 Motivational Example 1: The I‐SPY COVID Trial

### 3.1 Trial Description and Motivation

The I‐SPY COVID (NCT04488081) is a multicentre phase‐II open‐label adaptive platform trial aimed at identifying potential therapeutics for severe COVID‐19 [23] (Table A1). Patients with a positive SARS‐CoV‐2 test by PCR or rapid antigen testing on ≥ 6 L/min oxygen or intubated were screened for eligibility. Patients meeting the eligibility criteria were randomly assigned to either a common control arm, consisting of a backbone regimen of dexamethasone and remdesivir, or to backbone plus one of up to 4 investigational agents in the trial at any one time. Patients who provided informed consent were included in the modified intention‐to‐treat (mITT) population. The maximum number of patients who could be randomised to each investigational arm was 125.

The primary endpoints of the trial were time to durable recovery and time to death due to any cause. Time to recovery was defined as the time elapsed between randomisation and the earliest second consecutive day with WHO COVID level 4 (i.e., < 6 L/min oxygen) or below. In the analyses, follow‐up was censored at 60 days if patients were still at risk. An agent would graduate if the vector consisting of the posterior probability of the cause‐specific hazard ratio (csHR) for recovery being >1 and the posterior probability of the HR for all‐cause mortality being <1 was in the 2‐dimensional critical region $\left(\left[0.975,1\right)\times \left[0,1\right]\right)\cup \left(\left[0,1\right]\times \left[0.900,1\right)\right)$. Separate Bayesian Weibull models with weakly informative priors were used to model the cause‐specific hazard for recovery and the hazard for all‐cause mortality in the mITT population and to derive the posterior probabilities of graduation. These posterior probabilities were updated every 2 weeks and reviewed by an independent data monitoring committee (DMC). The primary role of the DMC was to ensure the safety of the patients in the trial, but also to recommend to the principal investigators that an agent should graduate or be dropped for futility, based on unblinded review of trial results. A detailed description of the study design, operating characteristics, and statistical analysis plan of the trial can be found elsewhere [23, 34, 35]. The analysis presented here replicates that performed at one of the interim reviews, with minor differences in statistical software and data, and it focuses solely on the time‐to‐recovery endpoint.

At the interim analysis approximately 15 weeks after the first patient was randomised to the backbone plus cyclosporine arm (2021‐07‐19), there were 58 patients in the cyclosporine arm and 75 in the control arm. A total of 34 patients in the cyclosporine arm and 47 in the control arm had recovered, while the corresponding numbers of patients who died before recovery were 7 and 12, respectively. The posterior median ${\text{csHR}}_{\text{recovery}}$ for cyclosporine versus control was 0.97 (95% quantile credible interval: 0.63, 1.47), indicating that the instantaneous recovery rate was similar in the two arms. The corresponding posterior probability of graduation was 0.44, outside the critical region $R=\left[0.975,1\right)$. Given that the interim data on the recovery endpoint was not in favour of cyclosporine after almost 50% of the maximum allowed number of patients had been randomised to cyclosporine, the DMC asked to predict the probability that cyclosporine would demonstrate sufficient benefit to meet the graduation criterion at the maximum sample size. The PPoS analysis was not prespecified in the trial protocol.

### 3.2 Results

Let the binary variable W be the baseline WHO COVID level of 6 or 7 (W=1) versus 5 (W=0) and the binary variable A be the cyclosporine (A=1) versus control arm (A=0). Due to randomisation, we assumed W to be independent of A. In the modelling phase, we specified a Bayesian model for the joint distribution of the competing event data, WHO COVID level, randomisation arm and the parameters, $p\left(t,x\;|\;w,a,\alpha ,\gamma ,\nu \right)p\left(w\;|\;\eta \right)\;p\left(a\right)p\left(\alpha ,\gamma ,\nu \right)p\left(\eta \right)$. The model $p\left(t,x\;|\;w,a,\alpha ,\gamma ,\nu \right)$ was specified in terms of the cause‐specific hazards for the two competing events, recovery from COVID (i=1) and death from any cause (i=2). In particular, we specified Weibull models stratified by randomisation arm so as not to impose any proportionality constraint between the cause‐specific hazards for the cyclosporine and the control arm: $\lambda_{\mathrm{ia}}\left(t\;|\;w,\alpha_{\mathrm{ia}},\gamma_{\mathrm{ia}},\nu_{\mathrm{ia}}\right)=u_{\mathrm{ia}}\nu_{\mathrm{ia}}t^{\nu_{\mathrm{ia}}-1}$. We let the scale parameter $u_{\mathrm{ia}}$ depend on baseline WHO COVID level, such that $\log\left(u_{\mathrm{ia}}\right)=\alpha_{\mathrm{ia}}+\gamma_{\mathrm{ia}}w$, while the shape parameter $\nu_{\mathrm{ia}}$ did not depend on any covariates. The following priors completed the model specification: $\alpha_{\mathrm{ia}}∼\text{Normal}\left(0,20\right)$, $\gamma_{\mathrm{ia}}∼\text{Normal}\left(0,\sqrt{0.5}\right)$, $\nu_{\mathrm{ia}}∼\text{Exponential}\left(1\right)$. The baseline WHO COVID level W followed a $\text{Bernoulli}\left(\eta \right)$ distribution with a noninformative prior $\eta ∼\text{Beta}\left(1,1\right)$. Lastly, we fitted the model to the interim data $d_{0}$.

In the prediction phase, we fixed the number of new patients in the cyclosporine arm to 125−58=67, and given that the randomisation probability to the cyclosporine arm was 0.45, we sampled the number of patients in the control arm from a $\text{NegativeBinomial}\left(67,0.45\right)$ distribution. For these new patients, we simulated the baseline WHO COVID level, time to event, and event type ($D_{\mathrm{new}}$). For the 33 patients still at risk at the interim analysis ($d_{\text{cens}}$), the outcome data were simulated conditional on the observed baseline WHO COVID level, randomisation arm, and their censoring times. Predicted follow‐up times were censored if they exceeded 60 days. The interim and predicted data were stacked in the dataset $D_{f}$.

In the analysis phase, we analysed interim and predicted data together using Bayesian proportional hazard models and then derived the posterior probability $\mathrm{Pr}\left({\text{csHR}}_{\text{recovery}}>1.00\;|\;D_{f}\right)$ over 4000 draws, as prespecified in the SAP. We repeated the prediction and analysis phase K=2500 times and approximated the PPoS as

$$\begin{array}{c} \text{PPoS}\approx \frac{1}{2500}\sum_{k=1}^{2500}1\left(\mathrm{Pr}\left({\text{csHR}}_{\text{recovery}}>1.00\;|\;D_{f}^{\left(k\right)}\right)\geq 0.975\;|\;{\tilde{\alpha }}^{\left(k\right)},{\tilde{\gamma }}^{\left(k\right)},{\tilde{\nu }}^{\left(k\right)},{\tilde{\eta }}^{\left(k\right)}\right)\\ \;=0.061, \end{array}$$

indicating that Cyclosporine would not likely have shown a benefit on the COVID recovery rate even at maximum sample size. Combining the information from the prespecified posterior probability analysis and the PPoS, the DMC recommended stopping randomisation of patients to the cyclosporine arm.

As the PPoS was low, we assessed in a sensitivity analysis how more enthusiastic priors about the ${\text{csHR}}_{\text{recovery}}$ for cyclosporine versus control for the remainder of the trial would affect the PPoS [36]. The rationale for this analysis was to reassure the DMC that their decision to stop the cyclosporine arm due to futility would be made even under optimistic assumptions about the effect of cyclosporine on the recovery rate in future patients. To this end, we specified the model $p\left(t,x\;|\;w,a,\alpha ,\beta ,\gamma ,\nu \right)$ so that the cause‐specific hazards for the two randomisation arms were proportional with a proportionality constant of $\exp\left(\beta_{i}\right)$. In particular, we defined $\log\left(u_{i}\right)=\alpha_{i}+\beta_{i}a+\gamma_{i}w$, $i=\left\{1,2\right\}$. The priors for $\alpha_{i}$, $\gamma_{i}$, and $\nu_{i}$ were defined as in the main PPoS analysis, while $\beta_{2}∼\text{Normal}\left(0,\sqrt{0.5}\right)$. We let the prior $\beta_{1}∼\text{Normal}\left(\mu ,\sigma \right)$ take on different values for the mean and standard deviation, covering different scenarios ranging from weakly neutral to strongly optimistic priors. We explored 15 different scenarios by setting $\mu =\left\{\log\left(1\right),\log\left(1.2\right),\log\left(2\right),\log\left(3\right),\log\left(4\right)\right\}$ and $\sigma =\left\{\sqrt{0.1},\sqrt{0.2},\sqrt{0.5}\right\}$. Some of these priors were extremely optimistic, for example the equal‐tailed 95% prior limits for the ${\text{csHR}}_{\text{recovery}}$ under the $\text{Normal}\left(\log\left(4\right),\sqrt{0.1}\right)$‐prior were $\left(2.15,7.43\right)$. These values were considerably larger than what was considered plausible at the time of the trial design, where the largest ${\text{csHR}}_{\text{recovery}}$ considered in the sample size calculations was 1.80.

**FIGURE 1:**
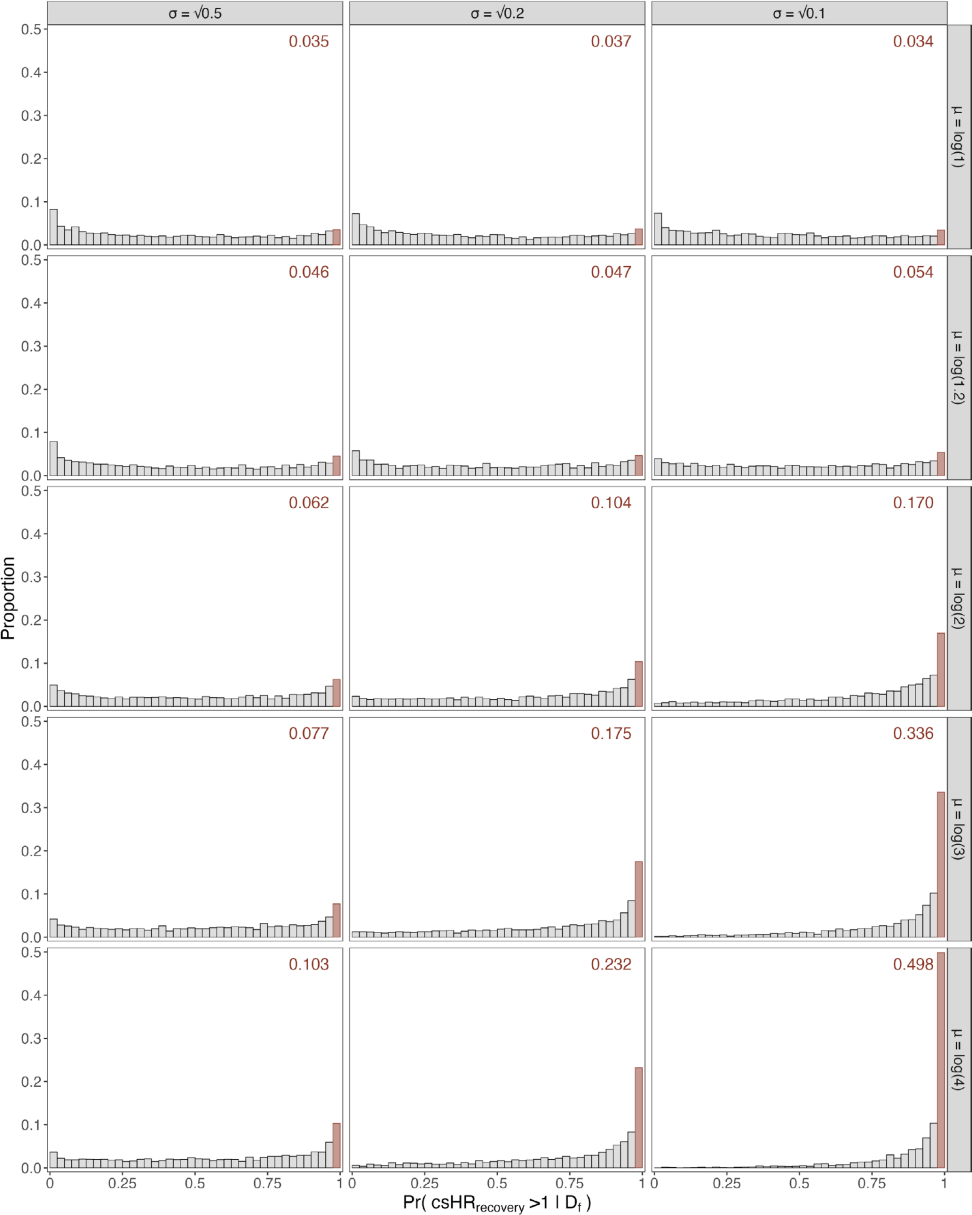
Distributions of the posterior probability of graduation $\mathrm{Pr}\left({\text{csHR}}_{\text{recovery}}>1.00\;|\;D_{f}\right)$ over 2500 simulations for the 15 different scenarios considered in the sensitivity analysis for the I‐SPY COVID trial. The red bar corresponds to the PPoS, i.e., to the proportion of simulations for which $\mathrm{Pr}\left({\text{csHR}}_{\text{recovery}}>1.00\;|\;D_{f}\right)\geq 0.975$. Each panel corresponds to a different prior $\beta_{1}∼\text{Normal}\left(\mu ,\sigma \right)$ specified in the modelling phase. The values specified for the prior's parameters μ and σ are reported along the rows and columns, respectively.

Figure 1 shows the distribution of the posterior probability $\mathrm{Pr}\left({\text{csHR}}_{\text{recovery}}>1.00\;|\;D_{f}\right)$ over 2500 simulations for the 15 different scenarios. The PPoS varied from 0.034 to 0.498, indicating that even an unrealistically optimistic and strong prior for ${\text{csHR}}_{\text{recovery}}$ resulted in a relatively low PPoS. This sensitivity analysis confirms that cyclosporine is unlikely to reach the graduation threshold for recovery at maximum sample size even under an extremely optimistic scenario.

## 4 Motivational Example 2: Long‐Term Follow‐Up of the STHLM3 Trial

### 4.1 Trial Description and Motivation

The population‐based diagnostic STHLM3 trial (ISRCTN84445406) was conducted between May 2012 and January 2015. A random sample of men aged 50–69 years living in Stockholm, Sweden, was invited to participate in a one‐time prostate cancer screening intervention using prostate‐specific antigen (PSA) and the Stockholm3 test, a prediction model based on clinical variables, plasma protein biomarkers, and a polygenic risk score [24]. One of the secondary aims of the STHLM3 trial was to evaluate the efficacy of screening invitation on long‐term prostate cancer mortality (PCM) in the presence of death from other causes as a competing event, which is the focus of the analysis reported here. The population eligible for random invitation to participate in the screening intervention consisted of 240,494 men. Of these, 169,621 (70%) were randomly invited and 59,088 (35%) participated.

An interim analysis was performed using follow‐up data until the end of 2018 to compare the crude cumulative risk of PCM between the two groups at 6.5 years after invitation [37]. Crude cumulative risks $F_{i}\left(t\right)$ were estimated using the Aalen‐Johansen estimator and the difference in PCM risk was summarised by a risk ratio (RR) with the non‐invited group as the reference. A *p*‐value for the two‐sided alternative hypothesis $H_{1}:\mathrm{RR}\neq 1$ versus the null $H_{0}:\mathrm{RR}=1$ was calculated as $p=2\cdot \Phi \left(-\left|\frac{\log\left(\mathrm{RR}\right)}{\mathrm{ASE}\left(\log\left(\mathrm{RR}\right)\right)}\right|\right)$, where $\mathrm{ASE}\left(\log\left(\mathrm{RR}\right)\right)$ is the asymptotic standard error for the $\log\left(\mathrm{RR}\right)$ and $\Phi \left(\cdot \right)$ is the standard normal cumulative distribution function. We considered a two‐sided alternative hypothesis because we could not rule out a priori that screening invitation might increase PCM risk. At interim analysis, the significance level $\alpha \left(6.5\right)$ was set to 0.02. During a maximum of 6.6 years of follow‐up, we observed 101 prostate cancer deaths in the invited group and 54 in the non‐invited group, while the estimated PCM risks were 8.6 and 13.2 per 10,000 men, respectively (Table 1). The $\mathrm{RR}\left(6.5\right)$ for PCM was 0.66 (98% confidence interval (CI): 0.41, 1.06), p=0.04, which favoured the invited group but did not reach formal statistical significance.

**TABLE 1:** Interim analysis results of the STHLM3 trial. Number of randomised men (*N*), deaths from prostate cancer (PCa), deaths from other causes and corresponding mortality risks for the invited and non‐invited randomisation groups.

Randomisation group	*N*	Deaths from PCa	Deaths from other causes	PCa mortality risk	Other causes mortality risk
Invited	169,621	101	6644	8.6 per 10,000 men	525 per 10,000 men
Not invited	70,873	54	2814	13.2 per 10,000 men	536 per 10,000 men

*Note:* Risks at the interim analysis were estimated using the Aalen–Johansen estimator treating the other event as a competing event.

The time point for the final comparison of PCM risk between the two invitation arms was prespecified to be between 10 and 15 years after study initiation. The PPoS was used to guide the selection of the specific time point for the final analysis (FA). We first predicted the probability of finding a statistically significant RR after 10 years given the interim data and then we repeated the prediction process five other times, adding 1 year at a time until a maximum of 15 years, thus allowing for more prostate cancer deaths to accumulate. The alpha level for the final analysis $\alpha \left(\mathrm{FA}\right)$ used in the PPoS estimation was set at 0.035 (critical region $R=\left(0,0.035\right]$) so that the overall type I error rate, considering both interim and final analyses, would be maintained at 0.05 [38]. A formal threshold for the PPoS was not prespecified in the original protocol and the decision on when to perform the final analysis was agreed upon at a consensus meeting that included statisticians and principal investigators of the STHLM3 trial.

### 4.2 Results

In the modelling phase, we specified a Bayesian model for $p\left(t,x\;|\;e,a,\beta ,\gamma \right)p\left(\beta ,\gamma \right)$, where the numeric variable E represents age at baseline (years), and the binary variable A represents the randomisation group: invited (A=1) versus non‐invited (A=0). As enrolment in the trial was completed, modelling of baseline covariates was not necessary. To model $p\left(t,x\;|\;e,a,\beta ,\gamma \right)$, we assumed piecewise constant cause‐specific hazards (PCH) for the two competing events, prostate cancer death (i=1) and death from other causes (i=2). We stratified the model by invitation group and included age at baseline as a covariate. The cause‐specific hazards were piecewise constant on $L_{i}$ time intervals defined by the boundary knots $q_{0}=0$ and $q_{L_{i}}=+\infty$ by the internal knots $q_{i}=\left\{q_{1},\ldots ,q_{L_{i}-1}\right\}$:

$$\begin{array}{l} \log\left(\lambda_{\mathrm{ia}}\left(t|e,\beta_{\mathrm{ia}},\gamma_{\mathrm{ia}}\right)\right)=\beta_{\mathrm{ia}1}I_{1}\left(t\right)+\beta_{\mathrm{ia}2}I_{2}\left(t\right)+\ldots +\beta_{{\mathrm{iaL}}_{i}}I_{L_{i}}\left(t\right)+\gamma_{\mathrm{ia}}e\\ =\sum_{l=1}^{L_{i}}\beta_{\mathrm{ial}}I_{l}\left(t\right)+\gamma_{\mathrm{ia}}e, \end{array}$$

where $I_{l}\left(t\right)=1\left(q_{l-1}<t\leq q_{l}\right)$ [39]. For the cause‐specific hazard for PCM, we chose $q_{1}=\left\{2,3,4,5\right\}$ years, while for the cause‐specific hazard for death from other causes we chose $q_{2}=\left\{0.5,1,\ldots ,5.5\right\}$ years, giving $L_{1}=5$ and $L_{2}=12$ time intervals, respectively. The interim data contained more information on other cause mortality than for PCM (Table 1), allowing a finer grid for the internal knots. We completed the models by specifying a lag‐1–autoregressive prior on the parameters for the baseline hazard (β) and a weakly informative prior on the parameter for baseline age (γ) (Modelling Strategy A in Table 2). We then fitted the model to the interim data $d_{0}$.

**TABLE 2:** Modelling strategies used in the modelling stage of the STHLM3 trial PPoS analysis.

Modelling strategy	Specification	Priors and hyperpriors	PPoS at 10.6 years	PPoS at 11.6 years	PPoS at 12.6 years	PPoS at 13.6 years	PPoS at 14.6 years	PPoS at 15.6 years
A	PCH model	$\beta_{\mathrm{ia}1}∼\text{Normal}\left(-10,20\right)$	0.783	0.826	0.856	0.864	0.885	0.892
	$L_{1}=5$, $L_{2}=12$	$\beta_{\mathrm{ial}}-\beta_{\mathrm{ia}\left(l-1\right)}∼\text{Normal}\left(0,\tau_{\mathrm{ia}}\right)$ for $l=2,\ldots ,L_{i}$						
	$q_{1}=\left\{2,3,4,5\right\}$	$\gamma_{\mathrm{ia}}∼\text{Normal}\left(0,\sqrt{0.5}\right)$						
	$q_{2}=\left\{0.5,1,\ldots ,5.5\right\}$	$\tau_{\mathrm{ia}}∼\text{Exponential}\left(1\right)$						
B	PCH model	$\beta_{\mathrm{ia}1}∼\text{Normal}\left(-10,20\right)$	0.758	0.796	0.832	0.868	0.877	0.892
	$L_{1}=L_{2}=3$	$\beta_{\mathrm{ial}}-\beta_{\mathrm{ia}\left(l-1\right)}∼\text{Normal}\left(0,\tau_{\mathrm{ia}}\right)$ for l=2,3						
	$q_{1}=q_{2}=\left\{2,4\right\}$	$\gamma_{\mathrm{ia}}∼\text{Normal}\left(0,\sqrt{0.5}\right)$						
		$\tau_{\mathrm{ia}}∼\text{Exponential}\left(1\right)$						
C	PCH model	$\beta_{\mathrm{ia}1}∼\text{Normal}\left(-10,20\right)$	0.783	0.825	0.850	0.863	0.884	0.894
	$L_{1}=L_{2}=5$	$\beta_{\mathrm{ial}}-\beta_{\mathrm{ia}\left(l-1\right)}∼\text{Normal}\left(0,\tau_{\mathrm{ia}}\right)$ for l=2,…,5						
	$q_{1}=q_{2}=\left\{2,3,4,5\right\}$	$\gamma_{\mathrm{ia}}∼\text{Normal}\left(0,\sqrt{0.5}\right)$						
		$\tau_{\mathrm{ia}}∼\text{Exponential}\left(1\right)$						
D	Weibull model	$\alpha_{\mathrm{ia}}∼\text{Normal}\left(-10,20\right)$	0.913	0.946	0.958	0.967	0.976	0.980
	Scale parameter: $\log\left(u_{\mathrm{ia}}\right)=\alpha_{\mathrm{ia}}+\gamma_{\mathrm{ia}}e$	$\gamma_{\mathrm{ia}}∼\text{Normal}\left(0,\sqrt{0.5}\right)$						
	Shape parameter: $\nu_{\mathrm{ia}}$	$\nu_{\mathrm{ia}}∼\text{Exponential}\left(1\right)$						

*Note:* PPoS at different time points for the final analysis are reported. Subscript i is the event type: i=1 death due to prostate cancer, i=2 death due to other causes. Subscript a identifies the randomisation group: a=0 not invited, a=1 invited.

Abbreviation: PCH, piecewise constant cause‐specific hazard.

In the prediction phase, we simulated the time to event and event type for those men who were still alive at the end of their follow‐up period in the interim data ($d_{\text{cens}}$), conditioning the predicted event time to be greater than the censoring time. To mimic the fact that the outcome data provided yearly by national registries cover the entire calendar year, the final analysis after 10 years will use data until the end of 2022. To this end, the predicted event times were censored at subject‐specific times given by the difference between 2022‐12‐31 and each subject's invitation date (i.e., time 0), giving a maximum follow‐up of 10.6 years. The same approach was applied for predictions at later time points. As enrolment in the STHLM3 trial is complete, the predicted data used in the final analysis $D_{f}$ consisted only of $d_{\mathrm{obs}}$ and $D_{\text{cens}}$.

**FIGURE 2:**
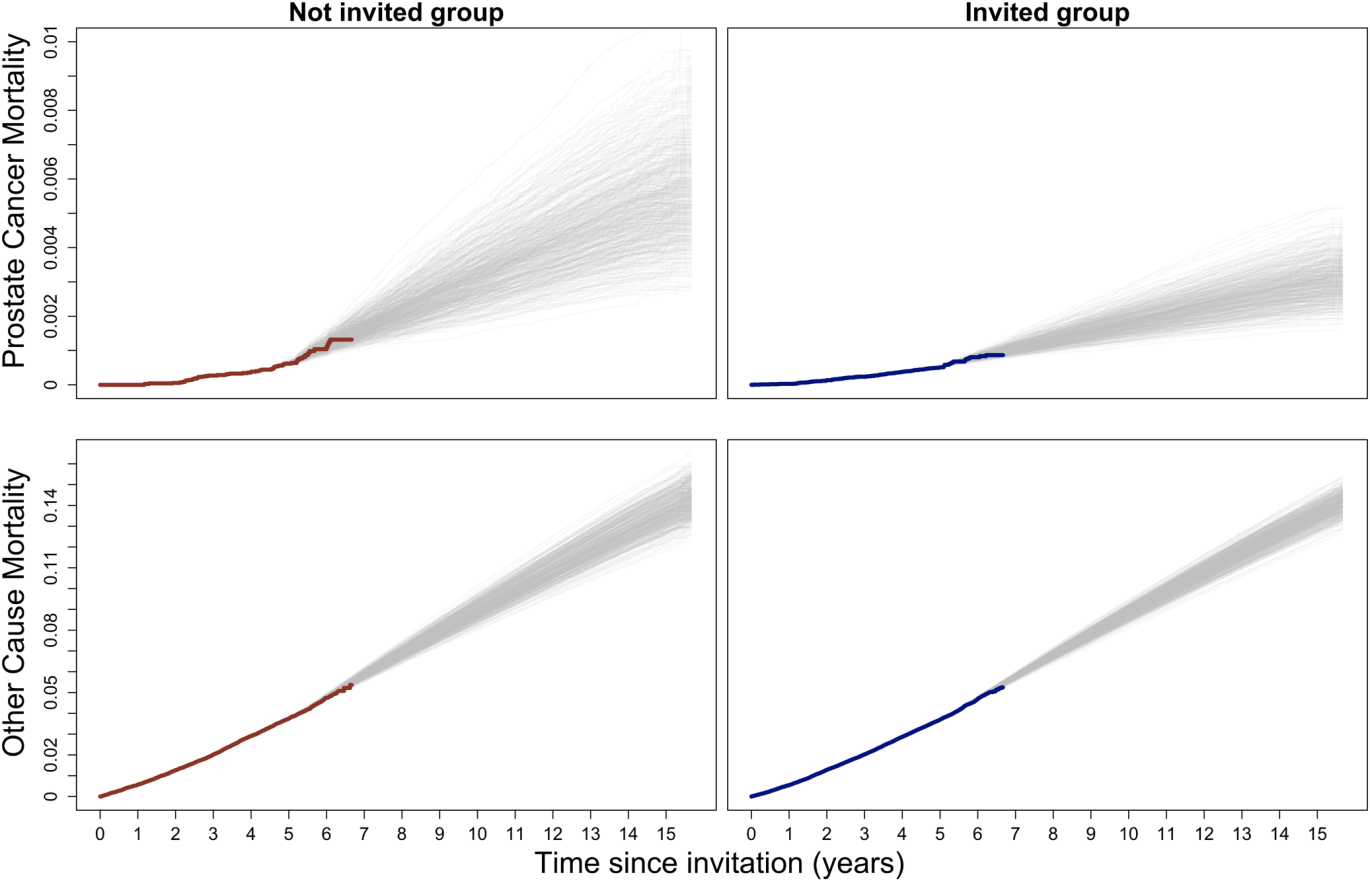
Red and blue thick step lines are the non‐parametric crude cumulative risks of death from prostate cancer and of death due to other causes estimated on interim data ($d_{0}$) for men invited and not invited to participate in the STHLM3 trial, respectively. Grey step lines are a random sample of 500 out of the 2500 non‐parametric crude cumulative risk estimates based on interim and predicted data together ($D_{f}$). Predicted data was simulated using Bayesian piecewise constant cause‐specific hazard models with 5 and 12 time intervals for death from prostate cancer and death from other causes, respectively (Modelling strategy A).

For each final analysis time point, we repeated K=2500 times the prediction and analysis phase. At each iteration, we estimated the cumulative PCM risks in the two invitation groups (Figure 2) and derived the RR together with the associated *p*‐value at the time of final analysis, corresponding to the largest follow‐up time ($\mathrm{FA}=\left\{10.6,11.6,\ldots ,15.6\right\}$ years). The PPoS was approximated as:

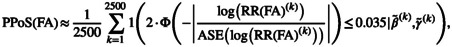

which ranged from 0.783 for the final analysis at 10.6 years of follow‐up to 0.892 for the final analysis at 15.6 years (Table 2, Modelling strategy A).

We performed a sensitivity analysis to assess the extent to which the choices made in the modelling phase could affect the PPoS. In particular, we first explored the consequences of changing the number and location of the internal knots of the PCH models, and then used Weibull models for the cause‐specific hazards. The specification of the alternative modelling strategies (B–D) is shown in Table 2 together with the corresponding PPoS, which ranged from 0.758 to 0.913 for the earliest time point for the final analysis (10.6 years), and was greater than 0.80 for later time points, regardless of the modelling strategy (Table 2). Since the PPoS was considered sufficiently high and robust across all modelling strategies for all time points, the statisticians and principal investigators of the STHLM3 trial decided that the PCM risk in the two groups would be compared at 11.6 years after invitation, i.e., with follow‐up data until the end of 2023.

## 5 Discussion

In this paper, we have described a simulation‐based approach to compute the PPoS for interim monitoring of clinical trials when the survival outcome is subject to competing events. Our work was motivated by two randomised clinical trials, both of which had a primary survival outcome with death as a competing event. In the I‐SPY COVID trial, the PPoS analysis complemented the prespecified efficacy analysis, which led the DMC to recommend stopping the randomisation in the backbone‐plus‐cyclosporine arm due to futility. In the STHLM3 trial, the PPoS analysis was used to inform the decision to perform the final analysis using follow‐up data covering until the end of 2023.

In the modelling phase, we have proposed the use of cause‐specific hazard models to model the joint distribution of time to event and event type given covariates. However, alternative approaches are possible and include multivariate latent failure time models [40], vertical models [41], and direct modelling of the cumulative incidence functions [42]. We have chosen cause‐specific hazard models because they provide a convenient and flexible modelling framework [43]. In addition, as the cause‐specific hazards allow the estimation of the joint distribution $\left(T,X\right)$ based on observable quantities alone, unverifiable assumptions about the dependence structure of the latent event times can be avoided. Different models can be specified to explore how sensitive the PPoS is to modelling assumptions, as we did in Section 4. However, in general, we recommend stratifying the cause‐specific hazard models over the randomisation groups, as done in previous applications [7, 14]. The use of baseline covariates other than randomisation arm can in principle improve the prediction of the outcome data [44]. This comes at the cost of an increased complexity due to the need to model their joint distribution, if new trial participants' baseline data are to be simulated. A simpler approach that however ignores some variability in the data generating process is to sample with replacement the observed baseline covariate data, i.e., from their empirical joint distribution.

The specification of the prior distributions represents a common concern with any approach based on Bayesian methodology, and the one proposed here is no exception. Weakly informative priors can be specified so that the predictions can be considered to be based on interim data only [2]. Informative priors may be otherwise specified to incorporate external information about the data‐generating process, including the treatment effect [45]. For PPoS sensitivity analyses, informative priors may be used to anticipate or explore the consequences of changes in the population or in the treatment effect over time [46], or to counterbalance the prior opinions of someone who would doubt the observed results [36], as we did in Section 3. Ultimately, the choice of the priors should be aligned with the objectives of the analysis. If necessary, the choice of the prior distributions can be discussed and agreed upon with the regulators at the time of trial design. Finally, it should be noted that the priors used in the modelling phase do not need to be the same as those used in the analysis phase.

As the proposed approach is simulation‐based, it can be computationally intensive, especially as the complexity of the prediction and analysis phase increases. Depending on the specific Bayesian models used in the modelling phase, predicting survival times can be a time‐consuming task. This is the case when the event‐free survival function cannot be inverted analytically or when it has to be evaluated by numerical integration [28]. The analysis phase can also be computationally demanding, especially when Bayesian methods are used. For reference, computing the PPoS for the I‐SPY COVID example took approximately 9 min on a high‐performance computing cluster using 40 cores, while for the larger STHLM3 trial it took over 10 h. The computational burden increases at the design stage, when the trial's operating characteristics are usually assessed for a possibly large number of scenarios. The number of simulations can be guided not only by time and computational constraints, but also by the accuracy with which the PPoS needs be approximated [47]. For example, our choice of K=2500 simulations was motivated by setting the upper bound on the standard error of the PPoS to 0.01. On the other hand, our approach offers full flexibility in the modelling and analysis phase and does not require resorting to asymptotic approximations [15] or to the use of conjugate priors [7]. In particular, the proposed framework can be applied to both frequentist and Bayesian trials, and any analytical method for competing event data—whether frequentist or Bayesian—can be used as the basis to define trial success in the analysis phase.

The use of Bayesian PPoS for interim monitoring of clinical trials is appealing because it is easily interpretable by trial stakeholders, including physicians, trial participants, and DMC board members. Furthermore, it directly addresses a relevant question: how likely is it that the trial will achieve its objective, given the current data, if it continues to its end? Importantly, this predictive question is addressed taking into account two sources of variability: the variability in the data not yet observed at interim monitoring and the uncertainty in the parameter estimates for the data‐generating process. At the same time, the calculation of the PPoS involves a number of assumptions that may lead to an inaccurate prediction of the success probability if they do not hold true [44, 46]. As we have done in our motivational examples, we recommend performing sensitivity analyses to evaluate how different assumptions may affect the PPoS [45]. The thresholds for the stopping rules that will trigger early termination of the trial are often specified at the design stage, including those based on the PPoS. This allows the trial's frequentist operating characteristics to be evaluated, for example using a simulation study, and the thresholds to be selected according to the desired long‐run properties of the resulting design [9]. Bayesian operating characteristics can also be used to select thresholds for early stopping in an analogous fashion [48].

In conclusion, our work extends the existing literature on the PPoS by proposing a simulation‐based approach that allows the PPoS to be used for interim monitoring of clinical trials in the common setting where the trial outcome is subject to competing events.

## Conflicts of Interest

M.E. reports four pending prostate cancer diagnostic‐related patents: method for indicating a presence or non‐presence of aggressive prostate cancer (WO2013EP7425920131120); prognostic method for individuals with prostate cancer (WO2013EP7427020131120); method for indicating a presence of prostate cancer in individuals with particular characteristics (WO2018EP5247320180201); and method for indicating the presence or non‐presence of prostate cancer (WO2013SE5055420130516). The Karolinska Institutet collaborates with A3P Biomedical in developing the technology for the Stockholm3 test. M.E. owns shares in A3P Biomedical. All other authors declare no conflicts of interest.

## Data Availability

The I‐SPY COVID trial data reported in this manuscript are maintained by the study sponsor, Quantum Leap Healthcare Collaborative (QLHC). Data can be shared with investigators external to the trial following approval by QLHC and the ISPY COVID Data Access and Publications Committee. This request can be initiated by contacting QLHC. The STHLM3 trial data reported in this manuscript are available from the corresponding author upon request after ethical approvals have been obtained from the Swedish Ethical Review Board.

## Appendix A

### COVID Executive Committee

Laura J. Esserman; Carolyn S. Calfee; Michael A. Matthay; Kathleen D. Liu; D. Clark Files; Martin Eklund; Karl W. Thomas.

**ARDS Executive Committee**

Laura J. Esserman; Kathleen D. Liu; D. Clark Files; Kevin Gibbs; Derek W. Russell; Martin Eklund; Karl W. Thomas.

**TABLE A1:** List of sites, principal investigators, and affiliations of the I‐SPY COVID consortium.

Site	Principal investigators (PI)	Affiliation
Columbia NYC	Jeremy R. Beitler, MD, MPH	Centre for Acute Respiratory Failure, Columbia University
Emory	Sara C. Auld, MD	Department of Medicine, Emory University
Georgetown	Nathan Cobb, MD	Georgetown University Medical Centre
Hoag (Irvine)	Philip Robinson, MD	Hoag Memorial Hospital Presbyterian Centre for Research and Education
Hoag (Newport Beach)	Philip Robinson, MD	Hoag Memorial Hospital Presbyterian Centre for Research and Education
KP LAMC	Kenneth K. Wei, MD	Division of Pulmonary and Critical Care Medicine, Kaiser Permanente Los Angeles Medical Center
Logan Health	Timothy Obermiller, MD	Critical Care Medicine, Logan Health Research Institute
Long Beach Memorial	Fady A. Youssef, MD	Department of Internal Medicine, Pulmonary Division, Long Beach Memorial Medical Centre
Main Line Health	Eliot Friedman, MD	Department of Medicine, Main Line Health
Mercy Hospital	Sandya Samavedam, MD	Critical Care Medicine, Mercy Hospital
Northwestern	Richard G. Wunderink, MD	Department of Medicine, Pulmonary and Critical Care Division, Northwestern University Feinberg School of Medicine
Sanford Health	Paul Berger, DO	Department of Critical Care, Sanford Health
Spectrum Health	Malik M. Khan, MD	Pulmonary and Critical Care, Spectrum Health
Stamford Health	Michael Bernstein, MD	Pulmonary Medicine, Stamford Health
U Penn	Nuala J. Meyer, MD	Department of Medicine, University of Pennsylvania Perelman School of Medicine
UAB	Derek W. Russell, MD	Pulmonary, Allergy, and Critical Care Medicine, University of Alabama, Birmingham
	Sheetal Gandotra, MD	Pulmonary, Allergy, and Critical Care Medicine, University of Alabama, Birmingham
UC Davis	Timothy Albertson, MD	Division of Pulmonary, Critical Care and Sleep Medicine, University of California Davis
UC Irvine	Richard A. Lee, MD	Division of Pulmonary Diseases and Critical Care Medicine, University of California
UCSF	Kathleen D. Liu, MD, PhD	Divisions of Nephrology and Critical Care Medicine, University of California San Francisco
	Laura J. Esserman, MD	Department of Surgery
	Carolyn S. Calfee, MD	Division of Pulmonary, Department of Medicine, Critical Care, Allergy and Sleep Medicine
	Michael A. Matthay, MD	Division of Pulmonary, Department of Medicine, Critical Care, Allergy and Sleep Medicine
University of Colorado	Ellen L. Burnham, MD	Department of Medicine, University of Colorado
University of Miami	Christopher P. Jordan, MD	Department of Pulmonary Care and Sleep Medicine
	Daniel H. Kett, MD	Department: Medicine, Division: Pulmonary, Critical Care and Sleep Medicine
University of Michigan	Robert Hyzy, MD	Division of Pulmonary and Critical Care Medicine, Department of Medicine, University of Michigan
USC	Santhi Kumar, MD	Division of Pulmonary, Critical Care and Sleep Medicine, Keck School of Medicine, USC
Wake Forest	D. Clark Files, MD	Department of Internal Medicine, Wake Forest University
	Kevin W. Gibbs, MD	Pulmonary Critical Care, Allergy and Immunologic Disease
West Virginia University	Jeremiah Hayanga, MD	Department of Cardiovascular and Thoracic Surgery, Heart and Vascular Institute, West Virginia University
Yale	Jonathan L. Koff, MD	Section of Pulmonary, Critical Care, and Sleep Medicine, Yale University
